# What are the views of cancer care administrators and clinicians in England on the use of a machine learning clinical decision support system (ML-CDSS) to predict patients’ risk of hepatic and renal deterioration during chemotherapy? A qualitative study

**DOI:** 10.1136/bmjopen-2025-107197

**Published:** 2026-05-18

**Authors:** Angelo Ercia, Mary Yip, Charlotte Reed, Luke Steventon, Clare Geoghegan, Matthew Watson, Christopher Kennelly, Pinkie Chambers, Noura Al Moubayed

**Affiliations:** 1 The University of Edinburgh, Edinburgh, UK; 2 Evergreen Life, Evergreen House, Clowes St, Manchester, UK; 3 Bennett Institute for Applied Data Science, Radcliffe Primary Care Building Radcliffe Observatory Quarter, Woodstock Rd, Oxford, UK; 4 University College London Hospitals NHS Foundation Trust, London, UK; 5 UCL School of Pharmacy, London, UK; 6 Department of Computer Science, Durham University, Durham, UK

**Keywords:** Machine Learning, Implementation Science, Clinical Decision-Making, Health Services, Pharmacists, Change management

## Abstract

**Abstract:**

**Objective:**

To explore administrators’ and clinicians’ views on the factors that influence their use and adoption of a machine learning clinical decision support system (ML-CDSS) to predict patients’ risk of hepatic and renal deterioration during chemotherapy.

**Methods and analysis:**

This was a qualitative study that used purposive sampling. 18 participants with administration and clinical backgrounds working in cancer care in England were recruited. Qualitative data were collected by conducting semi-structured interviews and a focus group. Data were analysed thematically using the framework method to identify key themes.

**Results:**

Participants acknowledged that monitoring blood chemistry is a core component of chemotherapy as it helps clinicians assess patient fitness and treatment response. The ML-CDSS was perceived as a potentially valuable tool for identifying patients at increased risk of hepatic and renal deterioration, supporting clinical decision-making and enhancing care efficiency. However, several concerns were raised regarding its potential implementation in practice. Participants questioned clinicians’ willingness and capacity to integrate the tool into their existing workflows. Participants also believed it was important to demonstrate the ML-CDSS’s sensitivity, specificity and validity in accurately predicting patients’ risk to build clinicians’ trust in the tool, demonstrating evidence of its efficacy and effectiveness in practice.

**Conclusion:**

Administrators and clinicians recognised the potential benefits of the ML-CDSS to enhance the delivery of chemotherapy by identifying patients at risk for hepatic and renal deterioration. Successful adoption in practice depends on building trust with the tool by being transparent in its development, its effectiveness and impact. Future work should demonstrate the ML-CDSS being used in practice to generate real-world evidence.

**STRENGTH AND LIMITATIONS OF THIS STUDY**

- The study approach enabled identifying factors that could impact the adoption and implementation of a machine learning clinical decision support system (ML-CDSS) into clinical practice.
- The study approach combined knowledge, expertise and experience from administrators and clinicians working in cancer care, providing diverse insights from key stakeholders that could influence the adaptation of using ML-CDSS in practice.
- The study combines interviews and focus groups to gain a more thorough and richer source of data.
- The study findings are not based on participants’ actual use and implementation of the ML-CDSS in practice.

## Introduction

 There were approximately 20 million new cancer cases and 9.7 million deaths caused by cancer globally in 2022^1^ with the incidence projected to rise to 26 million people by 2040.^2^ In the UK, about 393 000 people are diagnosed with cancer annually.^3^ An estimated 3 million people were living with cancer in the UK in 2020, which is expected to rise to 4 million by 2030.^3^ An ageing population, limitations in healthcare resources and growing socio-economic inequalities are some of the factors that impact cancer treatment outcomes.^4 5^ Many patients in the UK also face long waiting times to receive a diagnosis and/or treatment, which has been exacerbated because of an ongoing workforce shortage, resource constraints and residual impact of the COVID-19 pandemic.^3^,^5^ Survival rates, however, have risen steadily particularly in high-income countries like the UK and are partly due to advances in cancer treatments.^6^

Healthcare professionals treating patients with cancer are working in a world of dramatic innovation and increasing clinical complexity driven by genomics and immuno-oncology. Non-biomedical innovations from the advancement of Artificial Intelligence (AI) technology and its subset of Machine Learning (ML) are increasingly showing their potential impact in improving the delivery of quality and cost-effective treatment that enhances patient experience and outcomes.^5 7^ For example, AI technology enables the use of predictive analytics, leading to the practice of precision oncology in aiding the design of treatment.^5^ The technology can improve the efficiency of processing laboratory tests^5^ and support the timely administration of treatment as routine blood tests are required to assess the functions of the bone marrow, kidney and liver during treatment prior to each treatment cycle.^8 9^

While there are vast opportunities for AI to advance cancer treatment and its delivery, its use has yet to be widespread in clinical practice.^10^ Several reviews^11 12^ found that the implementation of AI technology in clinical practice is minimal. This project developed an ML-clinical decision support system (CDSS) for cancer care that could predict patients’ risk of hepatic and/or renal deterioration during treatment by analysing blood chemistry from cycles three and four for common chemotherapy regimens used to treat breast, colorectal and lymphoma cancers. The predicted outputs could reduce the number of blood chemistry tests for low-risk patients and provide better monitoring of higher-risk patients. To advance the progress of embedding AI technology in enhancing the delivery of chemotherapy, we aimed to explore administrators’ and clinicians’ views on the factors that could affect and facilitate the use of ML-CDSS in clinical practice.

## Materials and methods

This was an exploratory qualitative study that used interviews and a focus group. Semi-structured interviews with clinicians in England were selected because of its open-ended design to explore their views on using the ML-CDSS in practice.^13^

A focus group with administrators and clinicians working in cancer care across England was organised to discuss and build on ideas^13^ of the design and implementation of the ML-CDSS. Combining findings from the focus group and interviews provided a comprehensive understanding of the factors that could impact the use of the ML-CDSS at the micro and meso-level of delivering cancer care in the UK’s NHS system. This study was registered with the University College Hospital NHS Foundation Trust as a service evaluation. University College London (UCL) Research Ethics Services (REC) and NHS REC classify the project as a service evaluation and exempt from ethical approval.^14^

## Machine learning-clinical decision support software

UCL, Durham University and Evergreen Life collaborated to develop the ML-CDSS for this study. UCL and Durham University developed the ML model, and Evergreen Life developed the web-based platform that contained the ML model. Further description of the ML-CDSS and its development and performance evaluation can be found.^15^

### Inclusion criteria

The inclusion criteria for the interviews were clinicians (i.e., doctors, pharmacists, nurses) delivering chemotherapy at an NHS or non-NHS cancer care facility in England. The inclusion criteria for the focus group were administrators and clinicians responsible for commissioning, producing guidelines, resources and support in delivering cancer care.

### Participant recruitment

A purposive sampling approach was used to recruit 18 participants for the interviews and focus group.^16^ PC initially identified clinicians that would be appropriate to take part in the interview as she had an extensive network in cancer care and pharmacy due to her clinical and research background. The ‘snowballing’ sampling process was also used to identify other participants.^17^ Participants for the focus group were identified by PC and our project partner, UCL-Partners from their professional network in cancer care. There were no study participants that refused to take part or terminated their participation.

### Interview and focus group topic guide

The topic guide for the interview (online supplemental file 1) and focus group (online supplemental file 2) was developed by reviewing relevant literature, guidance from clinicians and guided by the Normalisation Process Theory by applying relevant constructs.^18^ The interview guide was semi-structured and covered topics including staff presence and activities in cancer centres, recording and discussing patient data, process of delivering chemotherapy, blood test collection and views on using the ML-CDSS. The structure of the focus group was designed with the research team. The focus group guide covered topics in understanding the enablers, barriers, external and internal factors that could affect the adoption of the ML-CDSS.

### Patient and public involvement

Clinicians contributed by developing and revising the interview guide. They also assisted with recruitment.

### Procedure

Participants for the interviews received an invitation email to take part and were instructed to reply if interested. The email described the aim and purpose of the interview. It included a brief description of the ML-CDSS as software that could be used in their computer desktop to analyse patients’ blood chemistry and identify their risk for hepatic and renal deterioration during chemotherapy. Team members (AE, MY) set up a time to conduct the interview either face-to-face on site or online using Microsoft Teams and answered questions or clarified any parts of the project. Eight interviews were conducted online and three were conducted face-to-face. The interviews occurred from July to October 2022 and lasted for 45–60 min. The interviews were conducted by AE (male) and MY (female). AE has doctoral level training in conducting qualitative studies. AE and MY have extensive experience conducting interviews in evaluation and research projects in digital health and healthcare systems. AE was an implementation manager and MY was a product manager for Evergreen Life. There were no repeat interviews conducted and data collection ended when the team agreed that data saturation was achieved^19^ after the 11th interview as participants were sharing similar viewpoints.

Focus group participants were invited through email describing the aim and purpose of the session, time, location and information about the ML-CDSS. Individuals wanting to take part were instructed to reply. The focus group was held in a 3-hour hybrid (face-to-face and online using Microsoft Teams) session in April 2023. All interviews and the focus group session were confidential, audio recorded with participants’ permission, transcribed verbatim and anonymised. Verbal consent was taken prior to commencing the interviews and focus groups. No field notes were made during the interviews or focus group as discussions were recorded. Transcribed transcripts were not returned to participants.

### Analysis

AE conducted the initial thematic analysis of the interviews and focus group using the Framework Method.^20^ An inductive approach was applied when developing the codes and themes. AE developed a coding framework used to code all the interviews. The coding framework included 25 distinct codes that identified activities, challenges and opportunities related to delivering chemotherapy and views on using decision support tools. Coding was completed manually using Microsoft Excel and Word. Once all the interviews were coded, initial themes were generated and discussed with MY and LB for feedback. Themes were then presented to the wider project team and discussed in multiple meetings to reach a consensus. Participants did not provide feedback on the themes.

## Results

There were 18 participants with diverse backgrounds who took part. A total of 11 participants were interviewed, and 7 participants attended the focus group (table 1). Interview participants were clinicians (doctors, nurses, pharmacists), with one participant holding both clinical and administrative roles. Participants in the focus groups were a mix of clinicians, clinicians with administrative roles and administrators.

**Table 1:** Professional role of participants in interviews and focus group

Interviews		Focus groups	
**Participant’s professional role**	**Number**	**Participant’s professional role**	**Number**
Doctor	1	Doctor	2
		Medical oncologist (1)	
		Clinical fellow (1)	
Nurse	4	Administrator	2
Clinical nurse specialist (1)		Project manager (1)	
Nurse divisional director (3)		Chair of Cancer Alliance group (1)	
Pharmacist	6	Administrator and patient	1
Pharmacist (5)			
Pharmacist/business development (1)			
		Administrator and pharmacist	2
		Senior programme manager/pharmacist (1)	
		Chair of Cancer Alliance group/pharmacist (1)	
Total	**11**		**7**

Understanding the chemotherapy clinical process and workflow was essential in determining how the ML-CDSS could be used and embedded in practice. Three themes were found about key elements of delivering chemotherapy. Another three themes were found related to understanding participants’ perspectives on pre-conditions for adopting ML-CDSS. Table 2 presents the themes and some direct quotes from participants.

**Table 2:** Themes and direct quotes from interviews and focus group participants

Key elements of delivering chemotherapy		
**Themes**		**Participant quotes**
1	Role of clinicians in delivering chemotherapy	*“The pharmacist screens at the same time. So once the [specialist doctor] approved [treatment], the pharmacist will then screen the prescription based on the bloods and the toxicity assessment and the dose, et cetera, et cetera.”* (Interview- Pharmacist/Admin 1)
2	Role of reviewing blood chemistry in chemotherapy	*“When they look at the patient’s bloods, they can see a trend, and it is unlikely that the patient would develop a renal failure. In the next one, but even so, you would still expect to do another blood test.”* (Interview- Doctor 1) *“You are really dependent on getting blood test results. For many other issues that your patients are experiencing.” *(Interview- Clinical Nurse Specialist 2 and 3)
3	Delays in reviewing blood chemistry	*“We probably end up chasing the majority of bloods after clinic.”* (Interview-Doctor 1)

**Participants’** perspectives on pre-conditions for adopting ML-CDSS		
4	Benefits of using the ML-CDSS	*“And I think would be a big advantage to patients if they could have it highlighted to them before it hits them.”* (Focus group- Admin/Patient 1) *“I would use it if I was supported, because it’s those red lines that you need a bit of reassurance and safety, that your decision is informed as opposed to a well…this patient looks like they've been fine all along.”* (Focus Group- Doctor 1) *“The potential benefits to pharmacy efficiencies, cost, medicines wastage, all those sorts of things. So, the appetite would certainly be there.”* (Focus group- Admin/Pharmacist 1)
5	Concerns for using the ML-CDSS	*“I suspect the nurses would find it quite difficult to accept and just from previous experience, they're very, very practical driven.”* (Interview- Pharmacist 3) *“The most important thing is covering the nurses, so they don't make any mistakes or anything like that, or if there are some kind of (recommended) deviations (to the standard practice it is recorded)…this machine learning model, you don't need to know the equation, you don't need to do all the algorithms, you just need to know this decision was made…and [everyone] agrees with (the recommendation).”* (Interview- Clinical Nurse Specialist 1) *“The only thing is the unpredictable nature is when a patient takes loads of Ibuprofen, and you just don't know what’s going on with their renal function…sometimes it is about why has their renal function suddenly gone up.”* (Focus group- Doctor 1) *“This tool will reduce contact with patients and that might be a reason that some patients may not want to opt-in if they some patients really value that face-to-face time.”* (Focus group- Admin/ Doctor 2)
6	Clinical evidence needed for the ML-CDSS to use in practice	*“It needs to have something that says this has been validated in lung cancer because…the medicines I use… have always been validated for use in these particular cancers. I need that reassurance.”* (Focus Group- Doctor 1) *“I still think health professionals generally don't really understand how these, how these things work.”* (Focus Group- Admin/ Pharmacist 2) *“Collect the data to demonstrate the impact and the savings that you know on patient time on pharmacy time, on efficiencies, because that will then support the evidence.”* (Focus Group- Doctor 1)

ML-CDSS, machine learning clinical decision support system.

### Theme 1: roles of clinicians in delivering chemotherapy

All NHS and non-NHS cancer providers in England had a team-based approach that included junior and specialist doctors, pharmacists and nurses to deliver chemotherapy. Each clinician had a specific role across the chemotherapy pathway. Specialist doctors primarily prescribed chemotherapy, obtained patient consent for treatment, oversaw treatment and delegated tasks to pharmacists and nurses to monitor patients’ progress.

Pharmacists supported the doctor in developing treatment plans which included treatment dosage. Some pharmacists developed treatment plans based on their experience, skill set and their hospital protocol. Most pharmacists assessed and monitored treatment progress, reviewed blood chemistry and other biomedical work and supported patients by answering questions during their treatment.

Nurses administered and monitored treatment and provided patient support. Nurses conducted the pre-assessments of patients, provided educational materials and resources once treatment consent was obtained by the doctor. Nurses routinely reviewed blood chemistry, patient fitness for treatment, administered chemotherapy and acted as a point of contact during treatment.

### Theme 2: role of reviewing blood chemistry in chemotherapy

All interviewed clinicians stated reviewing blood chemistry was an integral part of delivering chemotherapy, as it enabled them to assess patients’ level of fitness and treatment progress. All clinicians stated they were required by their current clinical protocol to review patients’ blood chemistry prior to administering treatment. However, the type of drugs prescribed in chemotherapy also determined the frequency of reviewing patients’ blood chemistry.

While some clinicians would be interested in reducing the frequency of assessing patients’ blood chemistry prior to treatment, this practice could be difficult to adapt. For example, if there were no indication that a patient was at risk for hepatic or renal dysfunction, standard protocol still required them to review blood chemistry prior to every treatment cycle.

Clinicians believed the usefulness of assessing blood chemistry as it enabled them to holistically review the patient’s health and aid in confirming whether the prescribed treatment was appropriate.

### Theme 3: delays in reviewing blood chemistry

Most clinicians acknowledged that laboratory processes caused delays in receiving blood chemistry results. A factor that could cause delays was the format of results received from laboratories. Some hospitals collected blood chemistry within their facility or had partner sites that could immediately share results within their electronic patient reported (EPR) system. However, other hospitals relied on external laboratories. The results may then be formatted differently and cumbersome to integrate within their EPR system.

### Theme 4: benefits of using the ML-CDSS

Clinicians identified a range of benefits using the ML-CDSS in their chemotherapy pathway. Most participants from the interviews and focus group believed it would be beneficial to use the ML-CDSS to identify patients who were at risk for hepatic and renal deterioration. Interviewed pharmacists believed it would be useful to know patients’ risk early on to appropriately monitor them throughout treatment. Some focus group participants believed using the ML-CDSS could improve people’s quality of life during chemotherapy by reducing the frequency of obtaining blood tests.

A second benefit of using the ML-CDSS was the additional information given to clinicians about their patients that could better inform and support their treatment decisions. A clinician emphasised that any digital tools that could help their decision-making process are useful because they constantly make decisions about patient treatment.

A third benefit of using the ML-CDSS was the potential to change the current chemotherapy pathway to make it more efficient. The ML-CDSS could improve triaging patient blood chemistry and follow-up appointments. By identifying patients’ risk, a clinician could dedicate more time with higher risk patients. It could also reduce or remove clinicians’ dependency on blood test results to make decisions, thus reducing delays in treatment. Many focus group participants also believed using the ML-CDSS could positively affect the wider NHS system by reducing healthcare costs by reducing treatment delays, drug/treatment waste, unnecessary patient hospital visits and required blood tests.

### Theme 5: concerns for using the ML-CDSS

A concern from most participants in the interviews and focus group was the capacity and interest of clinicians in using and embedding the ML-CDSS into their current chemotherapy pathway. Using the ML-CDSS would require clinicians to change their way of working and potentially their roles and responsibilities. For example, a lead pharmacist stated their nurses strictly adhered to written protocol and avoided deviations. It would be essential to assess how using the ML-CDSS would change their way of working. A clinical specialist nurse believed if the ML-CDSS were to be adapted, their entire unit would need to accept using it and establish clear processes that recorded the recommendation and instructions on next steps. Extensive training on using the ML-CDSS would also be required.

A second concern raised by interviewed and focus group participants was the sensitivity and validity of the ML model in detecting behavioural factors of patients that could influence their risk of hepatic and renal deterioration.

Last, focus group participants acknowledged that using the ML-CDSS could inevitably reduce patient-clinician contact. This could be negatively viewed by patients, especially if they expect regular face-to-face interaction during treatment.

### Theme 6: clinical evidence is needed for the ML-CDSS to use in practice

Focus group participants believed it was important to generate evidence on the efficacy and effectiveness of using the ML-CDSS in practice. It would be essential to show evidence that described the specificity of the model in accurately predicting patients’ risk for hepatic and renal deterioration. Clinicians also emphasised the need to provide evidence that showed the model had been tested against a range of drug regimens and/or cancer types.

Focus group participants believed it was important to generate evidence that described the ML-CDSS’s financial cost and resource savings and its impact on patients’ quality of life. It would be valuable to present evidence on the impact of using the tool on clinicians’ (doctors, pharmacists, nurses) time. Last, they emphasised the need for the ML-CDSS model to be built on patient data from various backgrounds to ensure it could be used among their diverse patient population across the UK.

## Discussion

This study explored administrators’ and clinicians’ views on using ML-CDSS that could stratify the risk of developing renal and hepatic deterioration among patients with cancer during chemotherapy. While there is some research^21^,^23^ that explored clinicians’ perspectives on using AI technology in clinical practice, there remains a gap in understanding the collective viewpoints of both administrators and clinicians who would be part of commissioning and implementing this technology in cancer care. The study findings identified several factors that may need to be addressed to increase the likelihood of adopting and embedding an ML-CDSS into practice.

### Changing the current chemotherapy pathways

Clinicians acknowledged the need to change their chemotherapy pathway to adopt the ML-CDSS into their current workflow. However, all clinicians acknowledged that changing any aspects in their workflow could be a barrier as staff members have become accustomed to it and required change. Current workflows are designed to follow national (ie, UK’s National Institute for Health and Care Excellence agency) and manufacturer guidelines that are supported by evidence-based and best practice. Some clinicians may also be uneasy in adopting new approaches that could go against current standard practices such as reducing the number of blood chemistry that patients receive during chemotherapy unless new protocols are established. Providing evidence that shows the ML-CDSS produces trustworthy recommendations will aid in building trust and acceptability among clinicians. Producing evidence that shows using the ML-CDSS improves clinical efficiency, cost-effectiveness and requires minimal resources to implement could also generate support.

The rapid and dynamic innovations in cancer treatment and its delivery, coupled with a constant rise in demand for cancer care, require the chemotherapy clinical pathway to routinely change. However, changes in pathways are challenging for cancer units and centres.^24^,^26^ A recent systematic review found that only 34.2% of CDSS tools have been used in clinical practice.^27^ Most studies that have explored the implementation of AI technology are trials or pilot projects with a short timeframe and parameters.^28^ While this provides insights on feasibility in a controlled environment, there are still uncertainties in how AI/ML-CDSS could be effectively integrated in cancer unit settings.^28^ The findings of this study reflect other study findings that highlighted a range of barriers that could impact the implementation of AI technology in clinical practice. This includes potentially new administrative burdens introduced by the AI technology.^24 29^ The usability of the tool and integration in the current clinical workflow are also a burden that can affect implementation.^30 31^ It is essential to build real-world evidence that includes case studies demonstrating these tools’ use in clinical practice by clinicians. Developing blueprints that describe how the ML-CDSS could be embedded within the chemotherapy pathway and used in different settings could also encourage administrators and clinicians to adopt them into practice.

### Potential approach to implementing ML-CDSS in practice

All participants acknowledged the potential value of including the ML-CDSS to predict patients risk for hepatic and renal deterioration and improve chemotherapy delivery. The information could enable clinicians to better allocate their time and resources more efficiently and tailor their approach to monitoring patients based on their level of risk. A patient representative acknowledged that the information would be useful for patients as it could inform them early on about their risk of developing hepatic and renal deterioration during chemotherapy. However, using ML-CDSS could reduce the interaction between clinicians and patients.

In realising the opportunity to improve the delivery of chemotherapy and patient management through the use of ML-CDSS, our study findings suggest the need to engage with clinical champions to start using it in practice. Pharmacists were viewed as the most appropriate clinicians to use the ML-CDSS because of their role in developing and guiding treatment plans with specialist doctors and, in some cases, prescribing the treatment. They perform routine reviews of patients’ blood chemistry during treatment and can suggest changes to it. Reviewing patient blood chemistry has become increasingly difficult because of constant delays in receiving blood chemistry results, clinicians needing to chase up results or results being formatted differently and inaccessible in the hospital’s electronic patient record system. The ML-CDSS has the potential to address this problem by reducing the number of blood chemistry tests collected from patients in between treatment cycles.

Several studies^32 33^ and a review^34^ have also suggested that pharmacists would benefit from using AI/ML-CDSS as it could provide information and guidance on cancer management, particularly in treatment selection and patient support to manage symptoms and side effects. Li *et al*^35^ study of using an ML model to predict the risk of cardiotoxicity among patients with colorectal cancer also found the usefulness of the technology among clinicians as it enabled them to identify risk factors to better manage and mitigate any problems from the treatment. Predictive analytics in healthcare have been found to be useful in allocating resources more efficiently, as it supports proactiveness in delivering patient care.^36^ It also enables the delivery of care to become more tailored to the patient, which can reduce medication errors, increase the efficacy rate of drugs and identify preventative measures.^34 37^ Heudal *et al*^38^ analysis of empirical studies and expert opinion suggests the importance of integrating AI technologies as a support tool that can further enhance shared decision making. When integrated within a patient-centred framework, these tools can improve communication between the clinician and patient that guides decision making.^39^ However, clinicians and patients need to become knowledgeable of the strengths and limitations of AI technologies to ensure they understand its capacities in providing individualised recommendations.^38^

### Reliability and validity of the ML-CDSS tool

Clinicians believed a barrier to using the ML-CDSS was not knowing its validity and sensitivity in accurately predicting patients’ risks of hepatic and renal deterioration during chemotherapy. As the ML-CDSS primarily relies on analysing the data from two cycles of blood chemistry, clinicians were concerned that behavioural factors (i.e., diet, physical activities, etc.) or other health conditions (i.e., co-morbidities) that were not included in blood chemistry reports could affect its recommendations. This can affect the trust of clinicians using the tool and its incorporation into practice. All participants acknowledged the importance of having evidence that showed the validity of the ML-CDSS model, and it could effectively and efficiently predict patients’ risk. This must be validated using data sets from patients with diverse backgrounds. Providing evidence that the tool is validated would facilitate the process of building trust in the tool among clinicians.

Clinicians need to build trust with AI technologies by observing their reliability and validity in producing useful information that could guide their decision-making to gain acceptance.^40^ Many studies that have investigated clinicians’ acceptance of AI technology and its use in clinical practice have emphasised the need to transparently describe the development of AI technology and its appropriate use based on the model’s reliability and validity.^38^ It has also been suggested that the development of AI technologies should be a collaboration between a diverse range of stakeholders that include developers, healthcare providers and policymakers to ensure it produces a usable tool within a real-world context.^41^

### Strengths and limitations

A strength of this study was including diverse views of participants with different professional roles and responsibilities in delivering cancer care across NHS and non-NHS providers in England. A limitation of this study is that the ML-CDSS was not implemented in any of the participants’ practice sites and therefore heavily relied on assumptions and hypothetical discussion of how it could be implemented. Future research in this topic should include views of stakeholders’ part of actual implementation of the technology. More stakeholders with views on commissioning and improving cancer care services should be further consulted. Additionally, other stakeholders such as developers, IT personnel in the NHS Trusts, programme managers and decision makers that fund the use of AI technology in hospitals should be included.

## Conclusion

This study explored the views of administrators and clinicians to identify factors that could impact the use of the ML-CDSS in delivering cancer care. A challenge in introducing ML-CDSS in practice is the need to determine how it could be embedded which would require changes in the current chemotherapy pathway. Demonstrating the validity and reliability of the ML-CDSS is essential in building trust and acceptability among administrators and clinicians. Increasing real-world evidence of implementing this tool across different settings would further help build participants’ confidence in using it in clinical practice.

## Supplementary material

10.1136/bmjopen-2025-107197 online supplemental file 1

10.1136/bmjopen-2025-107197 online supplemental file 2

## Data Availability

Data cannot be shared as it contains personal and senstive information.
