# Supplementary material for: What are the views of cancer care administrators and clinicians in England on the use of a machine learning clinical decision support system (ML-CDSS) to predict patients’ risk of hepatic and renal deterioration during chemotherapy? A qualitative study
Source: BMJ Open. 2026 May 18;16(5):e107197. doi: 10.1136/bmjopen-2025-107197 (PMC13185009; doi:10.1136/bmjopen-2025-107197)
Supplement: online supplemental file 1 [file bmjopen-16-5-s001.pdf]

Interview guide

Location: ____ **Date:**_________________________

Interviewee:____

Interviewer:____

**Staff presence and activities**<br>• What staff are present in the unit?<br>• What is their role?<br>• What activities are they involved in throughout a shift/clinic?
**Staff interactions**<br>• With whom do staff interact with (Other staff, patients non-clinical staff)<br>• When do they interact with others and for what?
**Recording of patient data**<br>• What staff are involved with recording patient data?<br>• What do staff record and when/where?<br>• What systems do they use to collect patient data (paper-based and /or electronic)

**Reviewing and discussing patient data**<br>• What staff are involved reviewing and discussing patient data?<br>• What do staff review/ discuss and when?<br>• What systems do they use for patient data?<br>• With whom do they discuss patient data with (Other staff, patients)?
**Physical space**<br>• Any objects or space that may affect the implementation of the product<br>• Advertisement/ patient communication
**Social, cultural factors to consider in the unit**<br>• Who makes decision in the unit<br>• Interaction between staff and distribution of responsibilities<br>• Staff champion- any staff keen to engage in new projects

Additional questions to ask clinicians

Prescribing/ electronic prescribing

- What staff is part of prescribing?
- Is this the same for all the trust in London?
- What type of training do staff receive?
- What systems do prescribers use to perform their task?
- What staff members do prescribers interact with to perform their task?

Scheduling

- What staff is involved with scheduling appointments for patients and blood tests?
- What does staff schedule appointments for?
- What are the challenges with scheduling?
- Are there any challenges with scheduling patients to get their blood test results?

Blood tests prior to chemotherapy

- Are there any problems with receiving blood test results in a timely manner?
- How many blood tests do patients tend to get (per month? Per year?)
- What are the blood test results used for?
- What staff is involved with scheduling blood test?
- Where do patients get their blood test done?
- Do patients get access to their blood test results?
- Who teaches the patients how to read their blood test results?
- Do you use a Care Machine to get report of full blood counts? Is this common among all trust now?

Chemotherapy nursing

- Who works with the chemotherapy nurses?
- Do they review the blood test results?

Organisation of multiprofessional team

- In addition to doctors, nurses, pharmacists, what other stuff members are involved in cancer treatment? What do they do?
- How do non-clinical staff support the clinical staff?

- Which staff members interact with patients during their treatment?
- Which other staff members (Clinical and non-clinical) are involved with treatment?
