# Supplementary material for: What are the views of cancer care administrators and clinicians in England on the use of a machine learning clinical decision support system (ML-CDSS) to predict patients’ risk of hepatic and renal deterioration during chemotherapy? A qualitative study
Source: BMJ Open. 2026 May 18;16(5):e107197. doi: 10.1136/bmjopen-2025-107197 (PMC13185009; doi:10.1136/bmjopen-2025-107197)
Supplement: online supplemental file 2 [file bmjopen-16-5-s002.pdf]

Focus group agenda and question guide

I. Participant introduction
II. Introduction to the project - This project is looking at predicting kidney and liver damage in cancer patients receiving chemotherapy
III. Project updates
IV. Presentation of the ML-Clinical decision support system
V. Focus group discussion

<u>Focus group questions</u>

1. Questions or any feedback around the pathway that we are just highlighting now.
   - Presentation of findings from validating the care pathway with Trust
2. How do you feel about the proposed pathway if you think that we are capturing all the information we need to capture here?
   - what will be the staff perception of the pathway if such a pathway it is to be implemented in the clinical practice.
3. Question: Do all chemotherapy regimens follow the same pathway or does the pathway always differ? Please explain.
4. Question: What are the key challenges and issues that we're going to face with implementing this tool in practice?
   - Where do you see challenges or differences in implementing this tool in your current workflow
   - Any important considerations with differences in ethnic background of the population?
5. Question: how do you see genomics playing a role especially with the cancer treatment?
6. Question: There are different thresholds of evidence you need to have before having an AI- digital tool adopted. Clinical decision support tool is the lowest we can have. I mean I'm not saying 0 but it's lowest depending on the predictive or the diagnostic where you have higher barriers to adoption. What is that level of evidence or what evidence specifically would you want, would you want to see in doing so, especially on the different scenario that we just explored?
7. Question: Would you be interested to use this when we have an early proof of concept to implement within your unit?
