# Supplementary material for: What are the views of cancer care administrators and clinicians in England on the use of a machine learning clinical decision support system (ML-CDSS) to predict patients’ risk of hepatic and renal deterioration during chemotherapy? A qualitative study
Source: BMJ Open. 2026 May 18;16(5):e107197. doi: 10.1136/bmjopen-2025-107197 (PMC13185009; doi:10.1136/bmjopen-2025-107197)
Supplement: Supplementary file 3 [file bmjopen-2025-107197.reviewer_comments.pdf]

PEER REVIEW HISTORY

BMJ Open publishes all reviews undertaken for accepted manuscripts. Reviewers are asked to complete a checklist review form and are provided with free text boxes to elaborate on their assessment. These free text comments are reproduced below.

ARTICLE DETAILS

Title (Provisional)

What are the views of cancer care administrators and clinicians in England on the use of a machine learning clinical decision support system (ML-CDSS) to predict patients' risk of hepatic and renal deterioration during chemotherapy? A qualitative study

Authors

Ercia, Angelo; Yip, Mary; Reed, Charlotte; Steventon, Luke; Geoghegan, Clare; Watson, Matthew; Kennelly, Christopher; Al-Moubayed, Noura; Chambers, Pinkie

---

VERSION 1 - REVIEW

Reviewer **1**

Name **Besculides, Melanie**

Affiliation **Icahn School of Medicine at Mount Sinai, Institute for Healthcare Delivery Science-Population Health Science and Policy**

Date **22-Aug-2025**

COI **None**

Understanding the perceived feasibility and utility of ML-based CDSS from end users is an incredibly important topic. All too often systems are created-and in many cases have high sensitivity and specificity for predicting outcome-only for adoption to be low. This paper could add to the literature (particularly in oncology), but it needs strengthening.

• Abstract line 27 influences should be influence

• Abstract results lines 42-44. The sentence "Participants believed it was important to demonstrate the ML-CDSS's specificity in accurately predicting patients' risk was essential to building clinicians' trust in the tool.." does not read correctly. Please reword the underlined words to better covey your message. Maybe delete 'was essential to'

• Abstract conclusion lines 46-47. Current wording sound like CDSS delivers chemotherapy. Reword to state actual role of CDSS (identify patients…) Similar statement in the intro line 100.

• Key message line 64 CDSS stands for clinical decision support systems (not software). You have it correct in the abstract.

• Intro line 79 'wit this' should be 'which is'. Please have someone not involved in writing the paper read the paper carefully for typos.

• Intro line 94 Define "It': AI, precision medicine?

• Intro "The objectives were to explore the chemotherapy pathway and the role of blood chemistry" Please explain this better. (addressing my next point may do this)

• Intro: It would be helpful in the intro to describe the CDSS (what it would do/be capable of-how you described it to people in your study) to help give a context for the later section on benefits of using the CDSS. There is some of this in the discussion and moving to the intro will help the reader understand the results.

• Intro lines 111 and 112. You conducted the focus groups with admin and physicians together? I am concerned with openness of respondents that have mixed roles that are potentially hierarchical. This may not be an issue in the UK and perhaps there was a synergy of ideas between the groups.

• Did the focus groups build on the interviews (or vice versa) or were they completely independent? Did you use the results from one to inform the next? Why the combination of methods (e.g., availability of staff, purposeful for X reason)? Its fine to do this, just explain the reason.

• Participant recruitment 'PC initially identified clinicians that would be appropriate to take part in the interview across England'. Please describe how they decided who was appropriate. Same comment for the focus groups.

• Interview guide and focus group guides: lines 151-153 revise last sentence define 'its adoption' (e.g., CDSS for predicting…)

• Patient and public involvement: are clinicians patients or the public? Maybe N/A is more appropriate (if permitted)?

• Participant recruitment and procedures: There is overlap in these two sections in how the interviews were conducted (e.g., in person, by Teams). Only state in one place.

• Procedure: give the numbers for interviews conducted in each way (in person, Teams)

• Procedure: please state training of those who conducted interviews (e.g., doctoral level training in qualitative methods) and relationship with those interviewed (e.g., no hierarchical power over..) See COREQ guidelines for reporting.

• Procedures: "Most interviews were conducted remotely and one-on-one". By remotely you mean not in the hospital? Typically when we say remotely we mean the interviewer and interviewee are not in the same place. Consider rewording/defining.

• Procedures, analysis: please describe data coding (methods, who conducted, software used) and at what point saturation was reached and how you determined (e.g., weekly meeting with research team about interview responses)

• Results: "There were 18 participants that took part in the interviews (11 participants) and the focus group (7 participants)" This sentence doesn't flow well. Please reword to say that 18 took part in the study; 11 participated in interviews, 7 participated in focus groups.

• Results: Consider stating upfront that X number of themes were identified and then number the themes in the text. Also consider a theme table that lists themes and quotes.

• Table 1. Consider renaming. Instead of 'background', perhaps 'role'

• Roles of clinicians in delivering chemotherapy: line 186 'consultants' are mentioned. Who are these? Were any of the people in the study consultants?

• Benefits of using the CDSS: line 246. I would not list the number of pharmacists (two) for several reasons including 1) When we use semi-structured guides, not everyone is asked every question 2) qualitative data do not need to be quantified 3) by listing two and the fact that they are pharmacists you are potentially allowing for identification. Choose a more vague word here and in later text.

• Clinical evidence: lines 332/33 'Including data from patients from diverse backgrounds to build the ML-CDSS was essential to ensure it could be appropriately used for a diverse population in the UK setting..' Did this idea come from the study participants? We definitely want diversity in the samples we use to build CDSSs so we don't exacerbate existing disparities, but only put this in results if it came from your participants. Otherwise you can note in the discussion and cite some papers.

• Clinical evidence lines 318-21 are essentially saying the same thing as lines 333-35.

• Discussion lines 374-76. I am unclear what this line means 'The findings of this study reflect other studies that highlighted a range of barriers that could…' as the findings of the research that is cited here (e.g., data security) were not raised by the study participants.

• Discussion 386 add 'potential' to usefulness as I don't think you actually implemented the CDSS. Also I am struggling with the language of 'delivering chemotherapy' again. Please either describe what this means or adjust the wording.

• Discussion line 394 Clinical champions have been effective for promoting CDSS use but did your study participants mention this?

• Strengths/ limitations: reword the first sentence. A study aim shouldn't be a strength. Remove the word aim and frame as diversity in roles of study participants or the inclusion of clinicians and non-clinicians. Then you state that need greater diversity of views. Maybe instead of that being a limitation it could just be stated as a place for future research?

• Conclusion line 456 Define 'this tool'.

**Reviewer**	**2**
**Name**	**Zimolzak, Andrew**
**Affiliation**	**Baylor College of Medicine, Medicine**
**Date**	**01-Oct-2025**
**COI**	**None**

General comments, strengths

This is a qualitative study examining healthcare professionals' perspectives on the use of machine learning to predict adverse effects of chemotherapy. The work is important because it is crucial not only to study the creation and technical requirements of ML tools, but also to translate them into practice. The framing of the problem of implementation in practice (lines 98-99, references 11 and 12) is well put. There is a very useful description of roles or job titles in Table 1, which is sometimes lacking in similar studies. Altogether, the work is well written and straightforward to understand.

Primary critiques

* Say more about what the participants were told about the ML system. Was anything presented apart from the interview guide and focus group questions? (E.g., were they shown positive predictive value, or sample ML predictions?)

* The paper presents relatively generic insights that participants may have had about any decision support system, even though the work is framed as a specific niche (specifically machine learning, and in the setting of chemotherapy, specifically risk prediction of two adverse effects). Do participants have concerns unique to ML? Are there nuances unique to chemotherapy, in contrast to other decision support? This general slant may be due to the methods. The interview guide mentions "implementation of the product" only once and is primarily oriented towards participants describing their current care environment. The focus group questions do prompt participants to share their perceptions, challenges, etc., regarding implementation. This concern of mine does not need to be changed in this manuscript, as it is not a threat to the validity of the results; however, it limits the novelty/impact of the work. A deeper exploration of how AI-CDSS applies to these specific niches could be an area for future work.

* In the results, be explicit about what themes you uncovered. Is there a final list of themes? Are they simply the subheadings in results: role of clinicians, role in reviewing labs, delays, benefits, concerns, clinical evidence? This was not clear from the text. I don't see the exact word "theme" used in the results.

* I'm not convinced the SQUIRE checklist is pertinent. There is no intervention per se. It's qualitative, and any impact, outcomes, or measures are only what the participants were asked to envision. This has already been mentioned as a study limitation. A more important point is that many of the SQUIRE items are not suited for this type of study. According to the EQUATOR network guidelines, a checklist such as SRQR or COREQ may be a better fit.

* What are clinicians meant to do with the risk levels output by the CDSS? The results describe (for example) that clinicians want to know patient risk "to appropriately monitor," or "support their treatment decisions." The only specific action I see mentioned is ordering fewer blood tests. Is there anything more than this? Is the current practice to check labs for every person before every treatment, irrespective of the chemotherapy regimen? For decision making: Would it affect the chemotherapy dose? Choice of agent/regimen? Changes to concurrent non-chemo meds? Etc.

Minor/optional points

* "wit" should be "with": line 79

* Is there anything more to say about next steps? What studies will these qualitative themes inform? In what specific way(s) could you/others demonstrate "the validity and reliability of the AI/ML-CDSS" to healthcare workers?

* 393,000 annually versus 3 million "diagnosed with" cancer sounds like incidence vs. prevalence, but this is not clear from the wording. "Diagnosed with" could also be interpreted as an incidence. Consider clarifying. Something like "3 million people were living with cancer...." (introduction section, line 79)

* If space allows, specify what cancers or what chemo agents this CDSS applies to. The original paper mentions bowel, DLBCL, breast, lung, and ovarian cancers. I am unsure which chemotherapy drugs were included in the original study. Some treatments for these cancers surely carry an increased risk of liver or kidney toxicity, whereas other treatments do not.

VERSION 1 - AUTHOR RESPONSE

Reviewer 1

**Reviewer's feedback**	**Authors' response**
Abstract line 27 influences should be influence	Changed influences to influence.
Abstract results lines 42-44. The sentence "Participants believed it was important to demonstrate the ML-CDSS's specificity in accurately predicting patients' risk was essential to building clinicians' trust in the tool.." does not read correctly. Please reword the	We agree that the wording in this sentence is awkward and have changed the sentence by removing 'was essential to'. The revised sentence is now, *'Participants also believed it was important to demonstrate the ML-CDSS's specificity in accurately predicting*

underlined words to better covey your message. Maybe delete 'was essential to'	*patients' risk to build clinicians…'* lines 41-44.
Abstract conclusion lines 46-47. Current wording sound like CDSS delivers chemotherapy. Reword to state actual role of CDSS (identify patients…) Similar statement in the intro line 100.	We agree that the current working needs to change as it suggests the CDSS delivers chemotherapy. We changed this sentence to state *'Administrators and clinicians recognised the potential benefits of the ML-CDSS to enhance the delivery of chemotherapy by identifying patients at risk for hepatic and/or renal deterioration'*. lines 45-47<br><br>The sentence in lines 98-103 is also changed to describe the actual role of the ML-CDSS.
Key message line 64 CDSS stands for clinical decision support systems (not software). You have it correct in the abstract.	Removed the term 'software'.
Intro line 79 'wit this' should be 'which is'. Please have someone not involved in writing the paper read the paper carefully for typos.	Changed the grammar mistake of 'wit this' to '*which is'*
Intro line 94 Define "It": AI, precision medicine?	Changed 'It' to using the term 'The technology' to refer specifically to AI in line 92.
Intro "The objectives were to explore the chemotherapy pathway and the role of blood chemistry" Please explain this better. (addressing my next point may do this)	We addressed this by providing a description of the CDSS that provides context to the AI tool (next point)- found in lines 98-103. As the reviewer suggested, this would help provide clarity.
Intro: It would be helpful in the intro to describe the CDSS (what it would do/be capable of-how you described it to people in your study) to help give a context for the later section on benefits of using the CDSS. There is some of this in the discussion and moving to the intro will help the reader understand the results.	We agree that a better description of the CDSS and its capabilities is needed. We included a description of the CDSS and its aim functionality. This change can be found in lines 98-103:<br><br>*'This project developed an ML-clinical decision support system (CDSS) for cancer care that could predict patients' risk of*

	hepatic and/or renal deterioration during treatment by analysing blood chemistry from cycles three and ….'
Intro lines 111 and 112. You conducted the focus groups with admin and physicians together? I am concerned with openness of respondents that have mixed roles that are potentially hierarchical. This may not be an issue in the UK and perhaps there was a synergy of ideas between the groups.	Yes, we conducted the focus group with administrators and physicians to support a synergy of ideas. It was important for both groups to discuss the use of ML-CDSS in clinical practice as implementing it relied on both stakeholders sharing their perspective on its value, concerns, barriers, and opportunities. While we understand the potential concern of power dynamics among participants, participants were from various organisations across England and did not work in the same organisation. We added a statement about all participants represented various healthcare organisations in England in lines 112-113.
Did the focus groups build on the interviews (or vice versa) or were they completely independent? Did you use the results from one to inform the next? Why the combination of methods (e.g., availability of staff, purposeful for X reason)? Its fine to do this, just explain the reason.	We added a clear explanation on the rationale of combining the focus group and interview findings by explaining that it would provide us a more comprehensive understanding of the factors that could impact the implementation of the ML-CDSS. We added this statement in lines 114-116.
Participant recruitment 'PC initially identified clinicians that would be appropriate to take part in the interview across England'. Please describe how they decided who was appropriate. Same comment for the focus groups.	We provided additional information about PC's professional background in cancer care and pharmacy that helped with the recruitment process. We added the statements in lines 133-135 and lines 136- 137.
Interview guide and focus group guides: lines 151-153 revise last sentence define 'its adoption' (e.g., CDSS for predicting...)	We changed 'its' to 'ML-CDSS' to be clear what we were referring to.
Patient and public involvement: are clinicians patients or the public? Maybe N/A is more appropriate (if permitted)?	Clinicians are still considered part of the patient and public involvement process as they are users of the ML-CDSS so it was essential to include them.
Participant recruitment and procedures: There is overlap in these two sections in how the interviews were conducted (e.g., in person, by Teams). Only state in one place.	We changed this section and combined the overlapping information. We removed information about the procedure from the recruitment section and placed this in the procedure section.

Procedure: give the numbers for interviews conducted in each way (in person, Teams)	We included this information by stating that 8 interviews were conducted online and 3 were conducted face-to-face. Found in lines 160-161.
Procedure: please state training of those who conducted interviews (e.g., doctoral level training in qualitative methods) and relationship with those interviewed (e.g., no hierarchical power over..) See COREQ guidelines for reporting.	We included the training and experience information for AE and MY who conducted the interviews in lines 162-164.
Procedures: "Most interviews were conducted remotely and one-on-one". By remotely you mean not in the hospital? Typically when we say remotely we mean the interviewer and interviewee are not in the same place. Consider rewording/defining.	We changed the term to online (line 160) rather than remote to ensure clarity.
Procedures, analysis: please describe data coding (methods, who conducted, software used) and at what point saturation was reached and how you determined (e.g., weekly meeting with research team about interview responses)	We included a description of the data coding and process of identifying data saturation. Additions can be found in lines 180-183.
Results: "There were 18 participants that took part in the interviews (11 participants) and the focus group (7 participants)" This sentence doesn't flow well. Please reword to say that 18 took part in the study; 11 participated in interviews, 7 participated in focus groups.	This sentence was reworded for clarity as suggested in lines 188-189.
Results: Consider stating upfront that X number of themes were identified and then number the themes in the text. Also consider a theme table that lists themes and quotes.	Thanks for this suggestion and we agreed this would further strengthen the presentation of our findings. We directly state the number of themes (line 195-196) in the Result section. We also developed a table that list all the themes and quotes (line 283).
Table 1. Consider renaming. Instead of 'background', perhaps 'role'	Changed the Table 1 title to "professional role of participants in interviews and focus group" for clarity in line 193.
Roles of clinicians in delivering chemotherapy: line 186 'consultants' are	Consultants are specialized doctors in the UK. For clarity, I removed the term

mentioned. Who are these? Were any of the people in the study consultants?	and replaced it with “specialist doctors” in line 201.
Benefits of using the CDSS: line 246. I would not list the number of pharmacists (two) for several reasons including 1) When we use semi-structured guides, not everyone is asked every question 2) qualitative data do not need to be quantified 3) by listing two and the fact that they are pharmacists you are potentially allowing for identification. Choose a more vague word here and in later text.	We removed the numerical value of two and referred to them as pharmacists (Lines 238-239).
Clinical evidence: lines 332/33 ‘Including data from patients from diverse backgrounds to build the ML-CDSS was essential to ensure it could be appropriately used for a diverse population in the UK setting..’ Did this idea come from the study participants? We definitely want diversity in the samples we use to build CDSSs so we don’t exacerbate existing disparities, but only put this in results if it came from your participants. Otherwise you can note in the discussion and cite some papers.	Yes, this was a finding from the focus group. To ensure this is clear, we moved this statement with the other findings from the focus group. This change can be found in lines 280-282.
Clinical evidence lines 318-21 are essentially saying the same thing as lines 333-35.	To remove redundancy, we restated the statement by removing the redundant statement of needing to generate evidence that demonstrated the ML-CDSS effectiveness in clinical practice.
Discussion lines 374-76. I am unclear what this line means ‘The findings of this study reflect other studies that highlighted a range of barriers that could…’ as the findings of the research that is cited here (e.g., data security) were not raised by the study participants.	This line aims to compare our study findings with others. We agree that data security was not raised by our study participants, so we removed this in our discussion.
Discussion 386 add ‘potential’ to usefulness as I don’t think you actually implemented the CDSS. Also I am struggling with the language of ‘delivering chemotherapy’ again. Please either	We agree and added the word “potential” (line 325). We also reworded the sentence to highlight the potential value of including the ML-CDSS to enhance

describe what this means or adjust the wording.	chemotherapy delivery and patient management.
Discussion line 394 Clinical champions have been effective for promoting CDSS use but did your study participants mention this?	Yes, our study findings suggest this, and we wanted to highlight it in our discussion. We did not suggest clinical champions have been effective for promoting the CDSS, but in our discussion, we suggest that clinical champions are needed to help encourage the tool's use in practice. NO change was made here. That said, we recognized that the title of this discussion point is inaccurate and changed it to "Potential approach to implementing ML-CDSS in practice" in line 324.
Strengths/ limitations: reword the first sentence. A study aim shouldn't be a strength. Remove the word aim and frame as diversity in roles of study participants or the inclusion of clinicians and non-clinicians. Then you state that need greater diversity of views. Maybe instead of that being a limitation it could just be stated as a place for future research?	We agree that needs to be changed. We removed the study aim as a strength. We stated a strength of the study was including a diverse group of participants representing clinicians and non-clinicians. We included a suggestion to ensure diverse participants representing different roles in delivering chemotherapy should be included in future research.
Conclusion line 456 Define 'this tool'.	We removed the term tool (line 391) and replaced it with ML-CDSS to be clear in what we are referring to.

Reviewer 2

Reviewer's feedback	Authors' response
This is a qualitative study examining healthcare professionals' perspectives on the use of machine learning to predict adverse effects of chemotherapy. The work is important because it is crucial not only to study the creation and technical requirements of ML tools, but also to translate them into practice. The framing of the problem of implementation in practice (lines 98-99, references 11 and	Thank you for your positive comments.

12) is well put. There is a very useful description of roles or job titles in Table 1, which is sometimes lacking in similar studies. Altogether, the work is well written and straightforward to understand.	

Primary critiques

Reviewer's feedback	Authors' response
Say more about what the participants were told about the ML system. Was anything presented apart from the interview guide and focus group questions? (E.g., were they shown positive predictive value, or sample ML predictions?)	We included more description on what the participants were told about the ML-CDSS as part of their invitation to take part in the interview and focus group in lines 155-158.
The paper presents relatively generic insights that participants may have had about any decision support system, even though the work is framed as a specific niche (specifically machine learning, and in the setting of chemotherapy, specifically risk prediction of two adverse effects). Do participants have concerns unique to ML? Are there nuances unique to chemotherapy, in contrast to other decision support? This general slant may be due to the methods. The interview guide mentions "implementation of the product" only once and is primarily oriented towards participants describing their current care environment. The focus group questions do prompt participants to share their perceptions, challenges, etc., regarding implementation. This concern of mine does not need to be changed in this manuscript, as it is not a threat to the validity of the results; however, it limits the novelty/impact of the work. A deeper exploration of how AI-CDSS applies to these specific niches could be an area for future work.	Thank you for raising this and we will certainly consider this for future work.
In the results, be explicit about what themes you uncovered. Is there a final list	Thank you for this suggestion and we agreed this would further strengthen the

of themes? Are they simply the subheadings in results: role of clinicians, role in reviewing labs, delays, benefits, concerns, clinical evidence? This was not clear from the text. I don't see the exact word "theme" used in the results.	presentation of our findings. We directly state the number of themes (lines 195-196) in the Result section. We also developed a table that list all the themes and quotes in line 283.
I'm not convinced the SQUIRE checklist is pertinent. There is no intervention per se. It's qualitative, and any impact, outcomes, or measures are only what the participants were asked to envision. This has already been mentioned as a study limitation. A more important point is that many of the SQUIRE items are not suited for this type of study. According to the EQUATOR network guidelines, a checklist such as SRQR or COREQ may be a better fit.	We applied the COREQ checklist and is now reflective in the narrative of the Methods section. We included the COREQ checklist as a supplementary document.
What are clinicians meant to do with the risk levels output by the CDSS? The results describe (for example) that clinicians want to know patient risk "to appropriately monitor," or "support their treatment decisions." The only specific action I see mentioned is ordering fewer blood tests. Is there anything more than this? Is the current practice to check labs for every person before every treatment, irrespective of the chemotherapy regimen? For decision making: Would it affect the chemotherapy dose? Choice of agent/regimen? Changes to concurrent non-chemo meds? Etc.	This is discussed in lines 238-240; 243-244; 248-249. We state that using the ML-CDSS will enable clinicians to identify patients risk level of developing liver and kidney damage. A patient with a higher risk may be allocated more time and attention to manage their condition.<br><br>A primary benefit of using the ML-CDSS is to potentially reduce blood tests. Standard practice requires blood chemistry to be checked prior to every treatment cycle which can be costly and as we described can experience much delays. Reducing the number of blood test for every treatment will help reduce unnecessary blood test, cost of blood tests, a reduce potential delays in decision making of administering the treatment.

Minor/ optional points

Reviewer’s feedback	Authors’ response
"wit" should be "with": line 79	We changed this.

Is there anything more to say about next steps? What studies will these qualitative themes inform? In what specific way(s) could you/others demonstrate "the validity and reliability of the AI/ML-CDSS" to healthcare workers?	We made further suggestions on future research studies on this topic.
393,000 annually versus 3 million "diagnosed with" cancer sounds like incidence vs. prevalence, but this is not clear from the wording. "Diagnosed with" could also be interpreted as an incidence. Consider clarifying. Something like "3 million people were living with cancer...." (introduction section, line 79)	We changed the wording to 3 million people were living with cancer to clarify the difference from the 393,000 people diagnosed annually.
If space allows, specify what cancers or what chemo agents this CDSS applies to. The original paper mentions bowel, DLBCL, breast, lung, and ovarian cancers. I am unsure which chemotherapy drugs were included in the original study. Some treatments for these cancers surely carry an increased risk of liver or kidney toxicity, whereas other treatments do not.	Unfortunately, due to limited word count, we cannot include this in the paper but will be considered for future projects. This topic is also specific to treatment and slightly diverge from the main aim of the paper of presenting clinicians' views on using ML-CDSS as a decision support tool in delivering chemotherapy.

VERSION 2 - REVIEW

Reviewer 1

Name Besculides, Melanie

Affiliation Icahn School of Medicine at Mount Sinai, Institute for Healthcare Delivery Science-Population Health Science and Policy

Date 17-Dec-2025

COI

Thank you for a thorough job revising your paper. I know it was a lot of work- it is much stronger and clearer as a result. My only suggestion is to match the Theme numbers in the text with those in the table. You have 6 in the text but in the table they restart.

VERSION 2 - AUTHOR RESPONSE

**Editors' comments**	**Response**

Reviewer 1: Thank you for a thorough job revising your paper. I know it was a lot of work- it is much stronger and clearer as a result. My only suggestion is to match the Theme numbers in the text with those in the table. You have 6 in the text but in the table they restart.	Thank you for identifying that unmatched number of themes in the table from the text. We changed the theme numbers in the table. Line 271-272<br><br>Thank you for acknowledging our revision based on your comments and other editors. We believe this revised version is much better. Many thanks for your time and effort to revise our paper.
Reviewer 2: If you have selected 'Yes' above, please provide details of any competing interests.: Not applicable	We have changed this answer and provided the following explanation in our paper. This information is also in the system. Line: 513-520.
